# Using the Chinese Version of the Screen for Disordered Eating to Assess Disordered Eating: Reliability, Validity and Correlates

**DOI:** 10.3390/healthcare13192458

**Published:** 2025-09-28

**Authors:** Tin Yan Cherry Cheung, Ming Yu Claudia Wong, Chak Hei Ocean Huang, Stanley Kam Ki Lam, Kadir Uludag, Ming Sing Jessica Choi, Shan-Yan Huang, Hong Wang Fung

**Affiliations:** 1 Department of Social Work, Hong Kong Baptist University, Hong Kong; 24481831@life.hkbu.edu.hk; 2 Department of Health and Physical Education, The Education University of Hong Kong, Tai Po, Hong Kong; cmywong@eduhk.hk; 3 Department of Social Work and Social Administration, The University of Hong Kong, Hong Kong; ocean9128@gmail.com; 4 School of Nursing and Health Sciences, Hong Kong Metropolitan University, Homantin, Hong Kong; skklam@hkmu.edu.hk; 5 Mental Health Center, School of Medicine, Shanghai Jiao Tong University, Shanghai 200030, China; kadiruludag@mails.ucas.ac.cn; 6 Department of Psychology, The University of Hong Kong, Hong Kong; jess102@hku.hk; 7 Department of Marketing and Supply Chain Management, Overseas Chinese University, Taichung 40721, Taiwan; 8 School of Nursing, The Hong Kong Polytechnic University, Hung Hom, Hong Kong

**Keywords:** screen for disordered eating, psychometric properties, reliability, validity

## Abstract

**Background/Objectives**: This study evaluated the psychometric properties and correlates of the Screen for Disordered Eating (SDE) in the Chinese context. Eating and body image in Asian cultures differed from those in the Western context, elevating its importance in testing the validity of SDE in the Chinese context. **Methods**: The reliability, validity and correlates of the SDE were examined in a sample of 766 university students in Taiwan. **Results**: Convergent validity of the Chinese version of the SDE was demonstrated with high correlation (r = 0.664, *p* < 0.001) and satisfactory consistency (κ = 0.413, *p* < 0.001) with the Sick, Control, One Fat, and Food (SCOFF) questionnaire. Substantial factor loadings were suggested, with 52% of the variance in item responses, especially for SDE4 and SDE5. Significant correlates of disordered eating were found with sex, depressive symptoms, anxiety symptoms, and experiences of childhood trauma. **Conclusions**: The SDE is a reliable and valid screening instrument for disordered eating. Health and social care practitioners can utilise the SDE to screen for disordered eating behaviours in the Chinese context, so that timely interventions can be provided.

## 1. Introduction

Disordered eating refers to unhealthy eating patterns, chronic dieting, and compulsive eating, which can lead to severe medical and psychological complications [1], such as anorexia nervosa, bulimia nervosa, and binge eating behaviour. Different from eating disorders, which are formally recognised in diagnostic manuals such as DSM-5 [2], disordered eating is an at-risk stage rather than an official diagnosis, which is essential for early detection and intervention. Disordered eating is widespread and affects people across different demographics [3,4], presenting significant challenges to public health in both Western and Asian populations. According to a global systematic review, meta-analysis, and meta-regression by [5], disordered eating is common among university students worldwide, with an average prevalence of 19.7%. Although there were no statistically significant differences between Western and non-Western countries, non-Western countries had a slightly higher weighted mean prevalence of disordered eating behaviour at 20.9% than Western countries at 18.4%. Specifically, the United States had a weighted mean prevalence of 37.1%, France reported 21%, China reported 4% and Saudi Arabia reported 21.2%. Previous literature emphasises that early detection using appropriate screening tools is crucial for managing the manifestations of disordered eating and implementing early interventions for improved prognosis [6]. Clinicians often have limited time to build rapport during initial screening [7]. In addition, disordered eating is a relatively sensitive topic, which is related to self-image, weight, and food [8]. Patients may be reluctant to disclose their conditions in interviews. A practical and short screening tool for disordered eating is essential for timely recognition and care.

To date, the five-item SCOFF, which stands for Sick, Control, One Fat, and Food, is one of the most widely used screening instruments for disordered eating [9]. Although it is generally used to identify probable disordered eating in individuals, rather than as a diagnostic tool in primary care settings, it has good sensitivity and validity, especially in assessing patients with mental disorders and young women’s disordered eating [10]. To address the influence of culture, an attempted adaptation of the Chinese SCOFF version was made with high-school students in Hong Kong [11]. Due to the simplicity and ease of administration of SCOFF, it is widely used in primary care and general population screening. However, the internal consistency of the Chinese SCOFF version has been reported as unsatisfactory due to its limitations in identifying cases of binge eating behaviour and other specified feeding or eating disorders (OSFED) [11,12].

To improve the effectiveness and accuracy of the diagnosis of disordered eating, the five-item screen for disordered eating (SDE) was developed as a more sensitive and specific screening instrument [12]. SDE aims to screen for disordered eating, including anorexia nervosa, bulimia nervosa and binge eating behaviour. Especially, binge eating behaviour had been overlooked in the SCOFF. However, the prevalence of binge eating behaviour in university students ranges from 0% to around 12.9%, depending on the populations studied and the tools used [13]. One survey studied over 3400 students, found 2.4% screened positive for binge eating behaviour and it was linked to depression and anxiety. Among them, females were reported to have higher rates. It has been utilised widely in primary care for diagnosis in Western countries [14]. In both SCOFF and SDE, a cutoff score of ≥2 is used to identify disordered eating. However, SDE performed better in identifying true cases overall, correctly detecting 87.2% of binge eating behaviour cases versus 69.6% for SCOFF [12]. SDE also has a higher sensitivity and specificity than SCOFF and the Eating Disorder Screen for Primary Care in diagnosing clinical eating disorders and differentiating non-clinical cases [15].

In particular, disordered eating may be milder in non-Western countries, which complicates the use of standard Western diagnostic tools, such as SDE [16]. Therefore, a culturally sensitive screening tool is important to be created to detect disordered eating in the Chinese population. In addition, clinical competence and empathy are required for medical professionals. However, it is difficult to address the above due to limited time and workforce, high workloads and administrative work [17]. An ideal consultation time, as suggested by patients, was around 15 min [18]. However, the consultation time was much shorter, especially in non-Western countries. For instance, it was around 6.8 to 8 minutes on average in Hong Kong, and around 3 min in Korea [19]. To address these issues, SDE requires relatively fewer clinical skills and training, offering a more convenient and instant detection, even out of hospital settings [10]. It also allows a better patient and caregiver experience and avoids delayed screening and diagnoses with early detection and early intervention in primary care settings.

Despite the superiority of SDE as a screening instrument, a Chinese version of it has not been adapted yet. Given that culture significantly influences attitudes toward eating and body image, it is crucial to test the adapted version of SDE with the Chinese population to ensure its validity in Chinese culture. Although SDE was designed for the primary care setting, it aims to identify individuals with potential eating disorders at an early stage. Studies of university students, which are community studies, can focus on higher-risk students with distinct stressors, for example, relationship issues, academic pressure that may potentially induce disordered eating [20]. Therefore, this study aims to validate the Chinese version of the SDE and explore its correlates. We hypothesised that SDE is a more reliable and valuable instrument to detect disordered eating compared to SCOFF and correlates with depression, anxiety and childhood trauma. This research is of paramount importance as it can facilitate the early identification of disordered eating among the Chinese population, thereby contributing to improved healthcare outcomes.

## 2. Methods

This study adopted a cross-sectional design to determine the reliability and validity of the Chinese version of the SDE in a sample of college students in Taiwan. This study was approved by the Office of Research and Development at the National Chung Cheng University, Chiayi, Taiwan on 12 August 2024 (CCUREC113070901).

### 2.1. Participants

We obtained ethical approval from the Human Research Ethics Committee of the National Chung Cheng University before the start of the study. Participants were recruited by completing an online mental health survey during National Chung Cheng University classes. Inclusion criteria included (1) being aged 18 or above; (2) being able to read and write Chinese; (3) being able to provide online written informed consent; (4) being a current university student. Exclusion criteria included (1) self-report having a medical diagnosis of a reading disorder, dementia, or intellectual disabilities. Students were invited by a co-author (a teacher at the university) to complete the online survey on Google Form during classes. Before they started filling out the survey, they read the information sheet of the study and provided an online written consent form on the first page of the Google Form. After completing the survey, respondents were given a small gift valued at around US$3.14 as a token of appreciation. The validity of the responses was checked by including the attention-checking items (e.g., 3 + 4 = ?). We excluded duplicate records from the analysis. Regarding data protection and confidentiality, no personally identifiable information was collected, except for the email address.

### 2.2. Measures

For demographic data, we collected the sex and age of the participants. Respondents completed the following standardised self-reported measures.

The SDE is a 5-item self-reported assessment for detecting disordered eating in mental health and/or primary care settings [12]. Participants completed the scale by indicating 1 = yes or 0 = no for each item. SDE was then computed with a cumulative score that ranges from 0 to 5. With a cutoff score of ≥2, the English version of the SDE demonstrated a sensitivity of 90.5% and a specificity of 57.5% [12]. Consequently, the threshold for the current Chinese scale version was established at 2 [21]. A collaborative translation approach was employed to ensure accuracy and cultural sensitivity of the Chinese version of SDE. In the present study, we used a collaborative approach [22,23,24] to translate the SDE into Chinese. In particular, two translators (a social worker and a nursing student) first independently translated the SDE into Chinese. The two Chinese versions were then compared, reviewed, and discussed in a panel of experts, including three PhD-level mental health researchers. The panel finally developed a Chinese version of the SDE used in the present study. The use of the collaborative approach was to ensure equivalence in meaning and concepts instead of the literal equivalence across languages [22].

The 5-item SCOFF was designed to detect disordered eating among primary care patients, adolescents, and university students [11]. SCOFF has been demonstrated to have good concurrent validity with DSM-IV diagnostic criteria for anorexia nervosa and bulimia nervosa [25]. The Chinese version of SCOFF has a good validity with 76.1% sensitivity (detecting disordered eating compared to EDE-Q) and 87.1% specificity in a sample of 812 secondary school students (response rate 86.8%) [11]. In addition, it has a moderate test–retest reliability (2-week interval, *n* = 38), ICC = 0.66. The internal consistency (Cronbach’s alpha) ranged from 0.44 to 0.57, acceptable for a 5-item scale. Participants were asked to answer five items with 1 = yes or 0 = no. A systematic review and meta-analysis of 25 studies [10] reported a pooled sensitivity of 86% and specificity of 83% for SCOFF. Previous research has adapted and validated the Chinese version [11].

Depressive symptoms were measured by the Chinese version of the 9-item Patient Health Questionnaire (PHQ-9) [26,27]. All items inquired about the severity of depressive symptoms participants have experienced in the past 2 weeks on a scale of 0 to 3. The four options were not at all = 0, several days = 1, more than half of the days = 2 and nearly every day = 3. The total score ranges from 0 to 27, with 0–4 indicating little to no degree of depression, 5 to 9 indicating mild depression, 10 to 14 indicating moderate depression, 15 to 19 indicating moderately severe depression and 20 and above indicating severe depression. The Chinese version of PHQ-9 was used [27]. It has a Cronbach’s alpha of 0.926.

Anxiety symptoms were measured by using the 2-item Generalised Anxiety Disorder (GAD-2) [28]. GAD-2 is scored by calculating the summative points of the questions. Responses were chosen from not at all = 0, several days = 1, more than half the days = 2, and nearly every day = 3. Participants had to rate how bothered they were by each anxiety symptom in the past 2 weeks. The study adopted the Chinese version of GAD-2 [29]. The Cronbach’s alpha for this measure was 0.882.

To evaluate childhood trauma, the 12 items from the Brief Betrayal Trauma Survey (BBTS) were used, which assessed 12 different types of traumatic events during childhood [30]. We defined a participant as being exposed to a specific traumatic event if they endorsed “one or two times” or “more than that” for an item. The test–retest reliability of the Chinese version of the BBTS was acceptable, with 90.7% (SD = 4.98) agreement one week apart [31].

### 2.3. Statistical Analysis

Using IBM SPSS 30 Software, we first evaluated the convergent validity of the Chinese version of the SDE by examining its correlation and agreement with the SCOFF. In addition to Pearson’s correlation analysis, we also examined the inter-rater reliability of the two questionnaires using Cohen’s kappa coefficient (Cohen’s κ) [32].

Psychometric properties of the scale were analysed utilising Item Response Theory (IRT) in R according to the nature of our dichotomous data. We adopted the MIRT package to run a two-parameter logistic model (2PL), considering each item’s difficulty and discrimination parameters. This analysis examined item discrimination parameters (‘a’) and item difficulty or location (‘b’). Items were deemed to exhibit a satisfactory fit when their information-weighted (infit) and outlier-sensitive (outfit) statistics ranged from 0.7 to 1.3, signifying optimal item efficacy.

**Figure 1:**
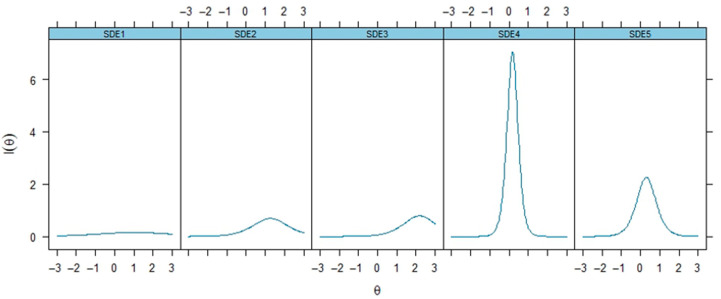
Item Information Curves.

**Figure 2:**
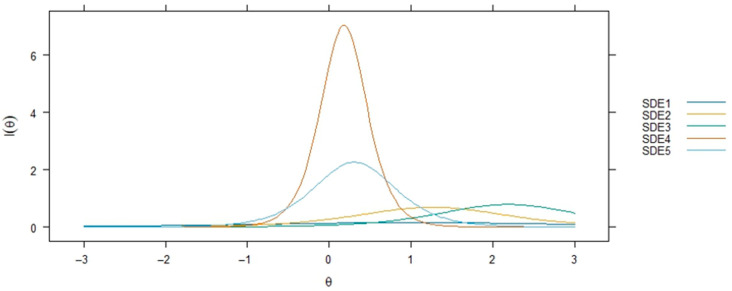
Item Characteristic Curves.

Item performance was represented with item information curves (IIC) (Figure 1) and item characteristic curves (ICC) (Figure 2). The IICs explained the amount of information contributed by each item throughout the entire range of the latent trait, with higher information values signifying better representation of the trait at various levels. The ICCs displayed the relationship between latent ability and item performance within each specified response category (1–5). Items with high peaks and spread over the ability continuum were identified as accurately assessing different ability levels.

Finally, logistic regression was conducted to examine the associations of the Chinese versions of the SDE and SCOFF with sex, age, depressive symptoms, anxiety symptoms, and childhood trauma.

## 3. Results

Seven hundred sixty-six students met the inclusion criteria, provided informed consent, and completed the survey from August to September 2024. Participants ranged in age from 18 to 30 years (M = 19.3, SD = 1.46); 56.9% were female, 67.4% were undergraduate students, and 4.4% reported seeing a psychiatrist in the past 12 months. Among the students who reported having seen a psychiatrist in the past 12 months, 26 reported having a diagnosed mental disorder. The other demographic characteristics are in Table 1. According to [33], a ratio of 10 participants per instrument item is often the “rule of thumb” for determining an adequate sample size for psychometric validation studies. The SDE was highly correlated with the SCOFF (r = 0.664, *p* < 0.001). There was moderate agreement between the SDE (≥2) and the SCOFF (≥2), κ = 0.413 (95% CI, 0.241 to 0.472), *p* < 0.001. These findings support the convergent validity of the Chinese version of the SDE.

In addition, the SDE’s exploratory factor analysis (EFA) revealed that a single-factor model explains a significant amount of variance (52%, SS loadings = 2.62) in the item responses. Each item demonstrated substantial factor loadings ranging from 0.41 to 0.92 (Item 1 = 0.41; Item 2 = 0.67, Item 3 = 0.62, Item 4 = 0.92 and Item 5 = 0.89), indicating a strong association with the latent factor. However, it is worth mentioning that a loading of 0.41 for SDE1 suggests that although it is significantly related to the factor, it has a weaker association than the other items in the set. However, using the general loadings above 0.4 is still considered acceptable. The model fit indicated through IRT shows that the Tucker–Lewis Index (TLI) and Comparative Fit Index (CFI) values were 0.895 and 0.92, respectively, slightly below the commonly accepted thresholds of 0.90 and 0.95 for adequate fit. The Root Mean Square Error of Approximation (RMSEA) was 0.10, with a 90% confidence interval of [0.08, 0.13], suggesting a marginal fit. The Standardised Root Mean Square Residual (SRMSR) was 0.066, indicating an acceptable residual fit. These indices suggest that the model captures some, but not all, of the underlying structure in the data, indicating a need for further refinement to improve model fit.

IRT parameters also provide detailed insights into the psychometric properties of the five items (SDE1–SDE5) through discrimination parameters (a), difficulty parameters (b1), infit and outfit mean square error (MSE) statistics, factor loadings, and communalities (h^2^), as presented in Table 2. Item loadings and commonalities (h^2^ values) from the model summary show that items SDE4 and SDE5 have very high loadings and explained variances, indicating that they are strong indicators of the latent trait measured by the scale. In contrast, SDE1 has a notably lower loading and explained variance, which is aligned with the EFA, suggesting it might be a weaker indicator. Also, referring to Figure 1 and Figure 2, SDE1, SDE2, and SDE3 show relatively flatter curves with lower peaks, suggesting they provide less information across the trait spectrum.

### 3.1. Difficulty Parameters

The difficulty parameters (b1) reflect the point on the latent trait continuum where the probability of endorsing an item is 50%. Item SDE3 (b1 = 2.1884) exhibits the highest difficulty, indicating that it requires a higher level of the latent trait for endorsement than other items. This suggests that SDE3 may target respondents with above-average levels of the construct. It could be appropriate if the instrument is designed to measure higher trait levels, but it may limit its utility for respondents with lower trait levels. Conversely, SDE4 (b1 = 0.1791) and SDE5 (b1 = 0.3081) have low difficulty parameters, indicating that these items are relatively easy to endorse, making them suitable for assessing respondents with lower levels of the latent trait. The range of difficulty parameters across items (from 0.1791 to 2.1884) suggests that the instrument covers a moderate span of the latent trait continuum, which is beneficial for broad measurement.

### 3.2. Discrimination Parameters

The discrimination parameters (a) indicate how effectively each item differentiates between respondents with varying levels of the latent trait. SDE4 (a = 5.3100) and SDE5 (a = 3.0075) exhibit exceptionally high discrimination, suggesting they are highly effective at distinguishing between individuals with different levels of the underlying construct. Such high discrimination values indicate steep item characteristic curves (ICCs; Figure 2), where slight differences in the latent trait lead to substantial changes in the probability of endorsing these items. In contrast, SDE1 (a = 0.7835) shows a relatively low discrimination parameter, suggesting it is less effective at differentiating between respondents across the latent trait continuum. This lower discrimination may contribute to reduced measurement precision for this item.

### 3.3. Item Fit Statistics

Infit and outfit MSE statistics provide valuable insights into how well each item meets the expectations of the Rasch model. Items generally show acceptable fit, especially SDE4 and SDE5, which have notably low misfit values. However, Infit and Outfit MSE values indicate that SDE1 might experience fit issues. It shows a high infit and outfit value (68.29), indicating noise or misfit beyond what is usually acceptable. This item’s poor performance also likely contributes significantly to the overall model misfit reported earlier.

### 3.4. Sampling Adequacy and Internal Consistency

The Kaiser–Meyer–Olkin (KMO) value of sampling adequacy was determined as 0.676, a moderate conclusion, and the factor analysis is appropriate (Table 3). Simultaneously, it has been determined that the test of Bartlett regarding sphericity was statistically significant (*x*^2^ = 665.457, *df* = 10, *p* < 0.001), demonstrating that the correlations observed in the data set were significant enough to allow the factor analysis. A value of 0.669 was obtained as the level of internal consistency between the five items of the scale, yielding an acceptable but moderate reliability coefficient as measured using Cronbach’s alpha.

Finally, logistic regression was performed to ascertain the effects of sex, age, depressive symptoms, anxiety symptoms, and total trauma numbers on the likelihood that respondents have disordered eating as measured by two scales, SDE and SCOFF (Table 4).

The SDE logistic regression was significant: χ^2^(6) = 147.957, *p* < 0.001. The model accounted for 23.5% of the variance in disordered eating (Nagelkerke R2), correctly classifying 68.4% of cases. Concretely, females were 2.514 times more likely to develop disordered eating compared with males. Increased depressive symptoms enhanced the likelihood of having disordered eating. The effect size of the SDE logistic model was 0.180, indicating a small effect size. In addition, the VIF values for the multicollinearity ranged from 1.062 to 3.232, suggesting low to moderate multicollinearity among the predictor variables.

Moreover, the SCOFF logistic regression model had a statistical significance of χ^2^(6) = 147.640, *p* < 0.001. It accounted for a 27.6% variance in disordered eating status (Nagelkerke R2), and the model classified 83% correctly. The odds of females manifesting disordered eating were 2.032-fold compared to Males. Increased manifestation of disordered eating is associated with increasing symptoms of depression and anxiety, as well as increasing childhood trauma experienced. The effect size of the SCOFF logistic model was 0.218, indicating a small to moderate effect. In addition, the VIF values for the multicollinearity ranged from 1.062 to 3.232, suggesting low to moderate multicollinearity among the predictor variables.

## 4. Discussion

This study explores the reliability, validity, and correlational relationships of the Chinese adaptation of SDE and SCOFF among Taiwanese university students. SDE exhibited a good correlation with SCOFF, attaining a Pearson correlation coefficient of r = 0.664 (*p* < 0.001), which signifies robust convergent validity. Moreover, SDE has a satisfactory consistency with SCOFF (κ = 0.413, *p* < 0.001), strengthening SDE’s ability to identify disordered eating. Exploratory factor analysis indicated that a single-factor model explained 52% of the variance in item responses, with substantial factor loadings for most items, especially SDE4 and SDE5. People with disordered eating may have a large desire to keep fit (related to SDE4 and SDE5) while being less concerned about their disordered eating behaviour (SDE1 to SDE3) [34]. This can explain the higher loading of SDE4 and SDE5. In addition, item response theory analysis indicates notable discrimination capabilities of these items, affirming their accuracy in identifying various levels of disordered eating. Lastly, the RMSEA value is relatively low, suggesting the model does not represent the observed relations closely. Item performance variability can explain this issue. The SDE1 that is related to emotional eating under stress may not align with the latent construct measured by other items.

Logistic regression analysis identified significant predictors of disordered eating, including sex, depressive symptoms, and experiences of childhood trauma. The prevalence of developing disordered eating in females was 2.514 times higher than in males. At the same time, symptoms of depression and experiences of childhood trauma were associated with a higher risk of disordered eating. These results highlighted the multifaceted nature of disordered eating and encouraged future disordered eating research to provide more insights into psychological and social factors associated with maladaptive eating behaviour [35]. In particular, clinicians can use SDE to effectively screen disordered eating among patients with mental disorders, especially for those with depression, anxiety and childhood trauma.

Although SDE displayed vigorous psychometric features, the exploratory factor analysis disclosed that SDE1 showed a considerably lower loading than other items and misfit among the items, denoting that it may be a less reliable indicator for disordered eating. To address this issue, there were several implications of the misfit statistics. First, SDE1 indicated emotional eating triggered by stress or confusion, which may not be specific enough to the pathology of the disordered eating. Many people without disordered eating may experience some stress-related changes in appetite [36], resulting in poor differentiation between cases and non-cases and thus this is not suitable for modelling. Second, disordered eating could be having no desire to eat in the face of emotional turmoil [36], such as anorexia. SDE1 may only capture disordered eating related to bulimia and binge eating behaviour, making it not a fit item to detect some of the disordered eating. Third, the SDE used unidimensional IRT in this study, which assumed items measure a single latent trait [37]. SDE1 primarily reflects emotional regulation rather than directly assessing disordered eating that may contribute to misfit statistically. Therefore, SDE1 was the relatively weak item among the other items. Clinicians can be aware of SDE1 during screening, noting that SDE1 may be the least representative for assessing disordered eating.

Regarding cultural validity, the Chinese version of SDE detected the unique cultural context of eating and body image in the Asian context. Due to sociocultural influences, including collectivism, media portrayals, and family dynamics, disordered eating and views toward body images may differ between Western and non-Western countries [38]. This difference suggested the importance of validating the Chinese version of SDE. Specifically, the higher loadings observed in the items of SDE4 and DSE5, on culturally significant food and body image items may reflect culturally normative patterns and social expectations regarding the expression of emotional eating. In contrast, the lower performance on SDE1 can be partially attributed to cultural norms that inhibit the open expression of emotions, contributing to differences in how young adults experience and report eating emotions. In addition, other instruments such as the Eating Attitudes Test (EAT-26) have shown strong psychometric properties in mainland Chinese populations but may not be practical for rapid screening [39]. Thus, considering the need to detect disordered eating with faster and more convenient screenings, we compared the Chinese version of SDE with SCOFF. The disordered eating interpreted by young adults in the Asian population may differ from that in Western countries due to cultural factors. For instance, emotional expression in different cultures may shape different responses, factor structures and societal attitudes toward food [40]. Although our findings show reliability, validity, and relevant correlates, including depression, anxiety, and childhood trauma (consistent with universal and culture-specific risk factors) of SDE, future studies should combine multiple screening measures and diagnostic interviews to establish cross-cultural validity and optimise screening accuracy further for different Chinese populations.

The items in SDE were explicitly designed to detect binge eating behaviours, making it particularly appropriate to be used among Asian populations, in which binge eating behaviours may be underreported or inadequately known [41]. SDE showed good accuracy in identifying different types of disordered eating, including more conventional diagnosis such as anorexia nervosa and bulimia nervosa. Thus, SDE can be considered a more holistic screening instrument than SCOFF. SDE is more appropriate for use in culturally diverse societies such as Taiwan, where the expression of disordered eating may be different from that of Western populations [42]. In Western countries, 13–20% of young adults were detected positive in disordered eating, suggesting a public health concern [43]. The SDE helped to identify the large at-risk group for early detection. Further, a holistic intervention can be implemented with the detected disordered eating and comorbidities. In Taiwan, the overall incidence and prevalence of disordered eating are lower than in Western countries. However, it is rising particularly for males. Most Taiwanese patients with disordered eating have psychiatric comorbidities, including depression (22%) and anxiety (53%) [44]. SDE could also assist clinicians in comprehending a patient’s challenges due to disordered eating and could provide insights into areas for developing more effective preventive strategies and interventions [45].

SDE has the potential to be used as a fundamental tool within mental health services settings, educational systems, and community health initiatives for the diagnosis or identification of disordered eating. For instance, clinicians may use SDE in standard screening with adolescents and young adults to facilitate the prompt detection of maladaptive eating patterns. By providing proactive measures, timely detection and interventions could be implemented in the community settings to mitigate the severe psychological and medical consequences frequently linked to disordered eating [46]. The timely detection and interventions can possibly reduce the strong stigma on disordered eating, which is particularly serious in the Asian context [47]. In addition, many Asian countries lack well-trained clinicians and professionals focusing on disordered eating [48]. The limited services and long waiting times may hinder early detection and interventions. The validated Chinese SDE reduces the likelihood of misclassification and provides a timely assessment and ease of use in clinical settings, which is helpful for less experienced clinicians [49]. This is also essential to avoid burdening the limited clinical resources with excessive follow-ups for false positives. The validated SDE also captured a wider range of disordered eating, which encouraged a more accurate identification of less common disordered eating (i.e., binge eating disorder) that were not considered in the SCOFF.

Although the findings highlighted the strengths of adapting SDE to Chinese settings, it is also essential to highlight its limitations. The first limitation is that SDE is a self-reported assessment, which may be prone to biases such as social desirability; additionally, a lack of self-awareness regarding eating habits could also yield inaccurate responses, threatening its validity. Second, although the typical age of eating disorder onset is 12 to 25 years old, this study mainly involved university students, which may limit the generalizability of the findings to the broader population, such as people in other age groups or youths who are at a similar age and are not studying in universities or vulnerable individuals (e.g., individuals at high risk of mental disorders). Future studies may seek to validate SDE with more diverse populations and settings to complement its applicability. This finding encouraged future studies to investigate the consistency and validity of each item in SDE instead of SDE to increase the precision of SDE in identifying disordered eating. Third, although some demographic characteristics were provided, some indicators relevant to disordered eating were not examined, such as, ethnicity, socioeconomic status, and BMI distribution. Future research can include these indicators better to understand the characteristics of people with disordered eating.

## 5. Conclusions

The Chinese version of SDE demonstrates substantial potential as a reliable and valid screening instrument for disordered eating among university students in Asian settings. Its ability to identify a spectrum of disordered eating, together with its strong psychometric characteristics, makes it a vigorous resource for clinicians to assess patients with a suspicion of disordered eating to facilitate the prompt detection of maladaptive eating patterns. By providing proactive measures, timely interventions could be implemented to mitigate the severe psychological and medical consequences frequently linked to disordered eating [46]. SDE could also assist clinicians in comprehending the challenges patients face due to disordered eating and could provide insights into areas for developing more effective preventive strategies and interventions [45]. By facilitating early detection and intervention, SDE can improve mental health outcomes and promote adaptive eating behaviour. Nevertheless, ongoing research and validation efforts remain crucial to addressing its limitations and confirming its sensitivity and validity across diverse populations.

## Figures and Tables

**Table 1:** Demographic characteristics.

	Sample Size	Percentage (%)
**Sex**		
Female	436	56.9
Male	327	42.7
**Educational level**		
Associate degree or equivalent level	240	31.3
Bachelor’s degree	516	67.4
Master’s degree and above	10	1.3
**Marital status**		
Married	3	0.4
Single	762	99.5
Divorced	1	0.1
**Seek help from mental health professionals**		
Never	675	88.1
Used to	77	10.1
currently	14	1.8
**having seen a psychiatrist in the past 12 months**	34	4.4
Diagnosed with physical issues	15	2.0
Diagnosed with mental health issues	36	4.7

**Table 2:** Infit and Outfit Statistics, IRT Parameters, and Model Fit.

Item	a	b1	Infit MSE	Outfit MSE	Loadings	h^2^
SDE1	0.7835	1.0260	68.29	68.29	0.418	0.175
SDE2	1.6577	1.2605	0.63	0.63	0.698	0.487
SDE3	1.7728	2.1884	1.31	1.31	0.721	0.520
SDE4	5.3100	0.1791	0.005	0.005	0.952	0.907
SDE5	3.0075	0.3081	0.002	0.002	0.870	0.757

**Table 3:** KMO and Bartlett’s test.

KMO measure of sampling adequacy		0.676
Bartlett’s test of sphericity	Approx. chi-square	665.457
	*df*	10
	Sig.	<0.001

**Table 4:** Results for Logistic Regression Models.

Variables	SDE ≥ 2	VIF	SCOFF ≥ 2	VIF
**Independent**				
Female	0.922 ***	1.062	0.709 **	1.062
Age	−0.040	1.043	0.034	1.043
Depressive symptoms	0.086 ***	3.160	0.070 *	3.160
Anxiety symptoms	0.005	3.232	0.331 ***	3.232
Childhood trauma	0.126	1.217	0.221 *	1.217
visited a psychiatrist in the past 12 months	−0.276	1.154	0.144	1.154
**Constant**	−0.738		−3.520 **	
***Nagelkerke*** (**pseudo R^2^**)	0.235		0.276	
***β***	0.180		0.218	
***N***	766		766	

*p* < 0.001 ***. *p* < 0.01 **. *p* < 0.05 *.

## Data Availability

The data set generated and analysed during the current study is available from the corresponding author (HWF) on reasonable request.

## Abbreviations

The following abbreviations are used in this manuscript:

SDE	Screen for Disordered Eating
SCOFF	Sick, Control, One Fat, and Food
IRT	Item Response Theory
IIC	item information curves
ICC	item characteristic curves
RMSEA	root mean square error of approximation
